# Functional Analysis of the *Borrelia burgdorferi bba64* Gene Product in Murine Infection via Tick Infestation

**DOI:** 10.1371/journal.pone.0019536

**Published:** 2011-05-03

**Authors:** Toni G. Patton, Gabrielle Dietrich, Marc C. Dolan, Joseph Piesman, James A. Carroll, Robert D. Gilmore

**Affiliations:** 1 Microbiology and Pathogenesis Activity, Division of Vector-Borne Diseases, National Center for Emerging and Zoonotic Infectious Diseases, Centers for Disease Control and Prevention, Fort Collins, Colorado, United States of America; 2 Tick-Borne Diseases Activity, Division of Vector-Borne Diseases, National Center for Emerging and Zoonotic Infectious Diseases, Centers for Disease Control and Prevention, Fort Collins, Colorado, United States of America; 3 Laboratory of Persistent Viral Diseases, Rocky Mountain Laboratories, National Institute of Allergy and Infectious Diseases, National Institutes of Health, Hamilton, Montana, United States of America; Charité, Campus Benjamin Franklin, Germany

## Abstract

*Borrelia burgdorferi*, the causative agent of Lyme borreliosis, is transmitted to humans from the bite of *Ixodes* spp. ticks. During the borrelial tick-to-mammal life cycle, *B. burgdorferi* must adapt to many environmental changes by regulating several genes, including *bba64*. Our laboratory recently demonstrated that the *bba64* gene product is necessary for mouse infectivity when *B. burgdorferi* is transmitted by an infected tick bite, but not via needle inoculation. In this study we investigated the phenotypic properties of a *bba64* mutant strain, including 1) replication during tick engorgement, 2) migration into the nymphal salivary glands, 3) host transmission, and 4) susceptibility to the MyD88-dependent innate immune response. Results revealed that the *bba64* mutant's attenuated infectivity by tick bite was not due to a growth defect inside an actively feeding nymphal tick, or failure to invade the salivary glands. These findings suggested there was either a lack of spirochete transmission to the host dermis or increased susceptibility to the host's innate immune response. Further experiments showed the *bba64* mutant was not culturable from mouse skin taken at the nymphal bite site and was unable to establish infection in MyD88-deficient mice via tick infestation. Collectively, the results of this study indicate that BBA64 functions at the salivary gland-to-host delivery interface of vector transmission and is not involved in resistance to MyD88-mediated innate immunity.

## Introduction

*Borrelia burgdorferi*, the causative agent of Lyme borreliosis, is transmitted through the bite of *Ixodes* spp. ticks, principally *Ixodes scapularis* in North America [1], [2], [3], [4]. The life cycle of *I. scapularis* is a 2-year process that contains three stages: larva, nymph, and adult. Ticks become infected with *B. burgdorferi* while consuming a bloodmeal from an infected reservoir host, typically small rodents. The resulting residential borrelial population adapts, through differential gene expression, to the feast and famine stages of the tick's enzootic cycle to be: 1) transtadially maintained in ticks, 2) transmitted to the vertebrate host, and 3) persistent in the reservoir vertebrate host [2], [5].

Numerous *in vitro* studies mimicking unfed and feeding tick environments have identified *B. burgdorferi* genes differentially regulated by changes in pH, temperature, cell density, carbon dioxide, and dissolved oxygen levels [6], [7], [8], [9], [10], [11], [12], [13]. Several borrelial genes that display pronounced differential expression in response to the altering environment are located on a 54-kilobase linear plasmid, termed lp54 [7], [9], [14], [15]. lp54 is one of the few plasmids found in *B. burgdorferi* that is consistently maintained in natural isolates [16], [17]. Encoded on lp54 are the proteins OspA, OspB, DbpA, DbpB, and CRASP-1 with described function and several other proteins with potential roles in borrelial pathogenicity [14], [15], [18], [19], [20], [21], [22], [23], [24]. Among this group of differentially expressed lp54 genes of undescribed function is *bba64*.

The *bba64* gene encodes an immunogenic 35-kDa lipoprotein localized on the borrelial surface [18], [25]. Expression of *bba64* was previously demonstrated to be regulated by RpoN-RpoS-Rrp2, BosR, and CsrA_Bb_ [26], [27], [28], [29]. *bba64* has been shown to be expressed in culture during stationary phase, during infection of the mammalian host, and within the tick after 72 hours of tick feeding; however, *bba64* is not expressed in flat (unfed) and 24 hour post-drop off replete ticks [15], [19], [27], [30], [31], [32]. The inactivation of *bba64* does not affect growth in culture medium, protein profiles, infectivity when *B. burgdorferi* is needle inoculated into mice, or xenodiagnosis [33], [34]. However, our laboratory recently demonstrated that a *bba64* mutant strain was attenuated in its ability to infect mice via tick infestation [33]. The aim of this study was to investigate *bba64*'s function pertaining to vector transmission to the vertebrate host. In this report, we temporally measured *bba64* transcription in the tick while feeding, and addressed whether the inability of the *bba64* mutant to infect mice by tick bite was due to: 1) a replication defect inside an actively feeding nymph, 2) impaired migration to the salivary glands, 3) failure to be transmitted into the mouse dermis during tick feeding, and 4) increased susceptibility to the host's Myeloid Differentiation marker 88 (MyD88)-dependent innate immune response.

## Results

### Total *bba64* expression during the nymphal bloodmeal

**Figure 1:**
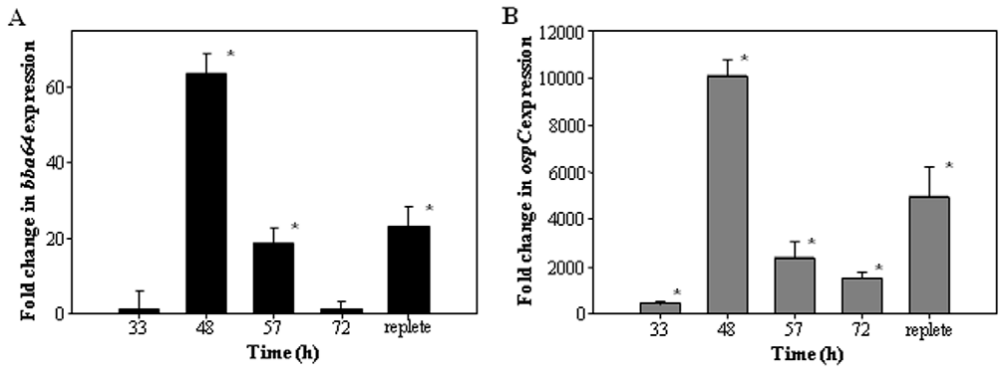
*bba64* and *ospC* expression during nymphal tick feeding. Fold change in A) *bba64* and B) *ospC* expression in actively feeding and replete WT-infected nymphs relative to flat nymphs. Data represents the average of 4 independent experiment samples per time point (each technically replicated in triplicate), and error bars correspond to the standard error of the means (SEM). Statistically significant differences (*) existed between baseline (0) and *bba64* or ospC expression (P<0.05) using Mann-Whitney rank sum test.

We assessed the *bba64* transcription profile throughout nymphal engorgement to temporally determine *bba64* expression relative to expression in WT-infected flat nymphs (0 h). Quantitative reverse transcriptase polymerase chain reaction (qRT-PCR) revealed that *bba64* expression was not detectable in the WT-infected flat nymphs, but was detectable 33 hours post nymphal infestation (Figure 1A). At 48 hours post nymphal infestation *bba64* expression was significantly upregulated (p = 0.010), approximately 60-fold, and remained significantly up-regulated at 57 hours (p = 0.004) (Figure 1A). Interestingly, *bba64* expression was not significantly increased at 72 hours post infestation, yet expression was significantly increased again in replete WT-infected nymphs, not more than 8 hours after nymphal drop off (p = 0.016) (Figure 1A).

As a positive control, we also measured *ospC* expression during nymphal engorgement (Figure 1B). qRT-PCR showed *ospC* was highly expressed throughout the nymphal feeding, as has been previously reported (Figure 1B) [35], [36]. The highest *ospC* expression was observed at 48 hours post infestation, revealing a 10^4^-fold increase relative to WT-infected flat nymphs (Figure 1B). Statistically significant differences existed between *ospC* and baseline expression (0) at all time points (33 h p = 0.021, 48 h p = 0.001, 57 h p = 0.037, 72 h p = 0.006, and replete p = 0.019).

### The *bba64* mutant replicates similarly to wild type (WT) in actively feeding nymphs

**Figure 2:**
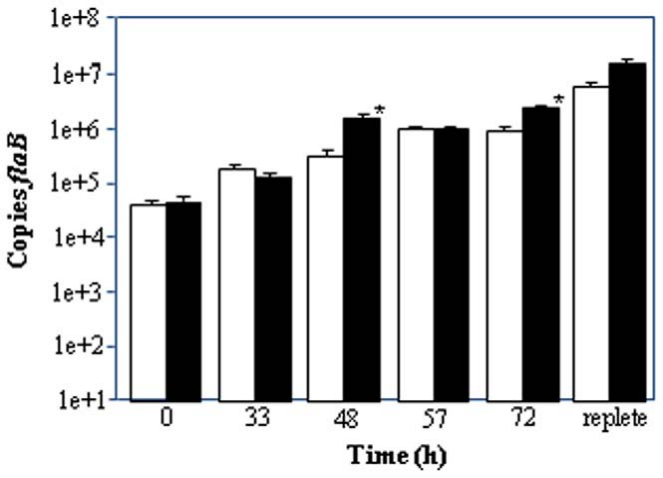
qPCR-based enumeration of *B. burgdorferi* in individual actively feeding nymphal ticks. WT- (white bars) or *bba64* mutant- (black bars) infected nymphs were fed on mice and removed at 33 h, 48 h, 57 h, and 72 h. Flat (0 h) and replete infected nymphs were also collected. *Borrelia* were enumerated by qPCR using *flaB* Taqman probe and primers. The data represents the average of 5 individually crushed nymphs per time point and error bars correspond to the SEM. Statistically significant differences (*) existed between WT and *bba64* mutant samples (P≤0.001) using Mann-Whitney rank sum test.

To ascertain if the *bba64* mutant's attenuated infectivity by tick transmission was due to a replication defect in actively feeding nymphs we measured total borrelial cell density in individual nymphs using qPCR. The numbers of spirochetes increased exponentially throughout the feeding for both WT and mutant organisms (Figure 2). No statistically significant differences existed in quantities between WT and the *bba64* mutant at all time points, except 48 h and 72 h; when there were significantly more *bba64* mutant spirochetes than in WT-infected nymphs (P≤0.001) (Figure 2).

**Figure 3:**
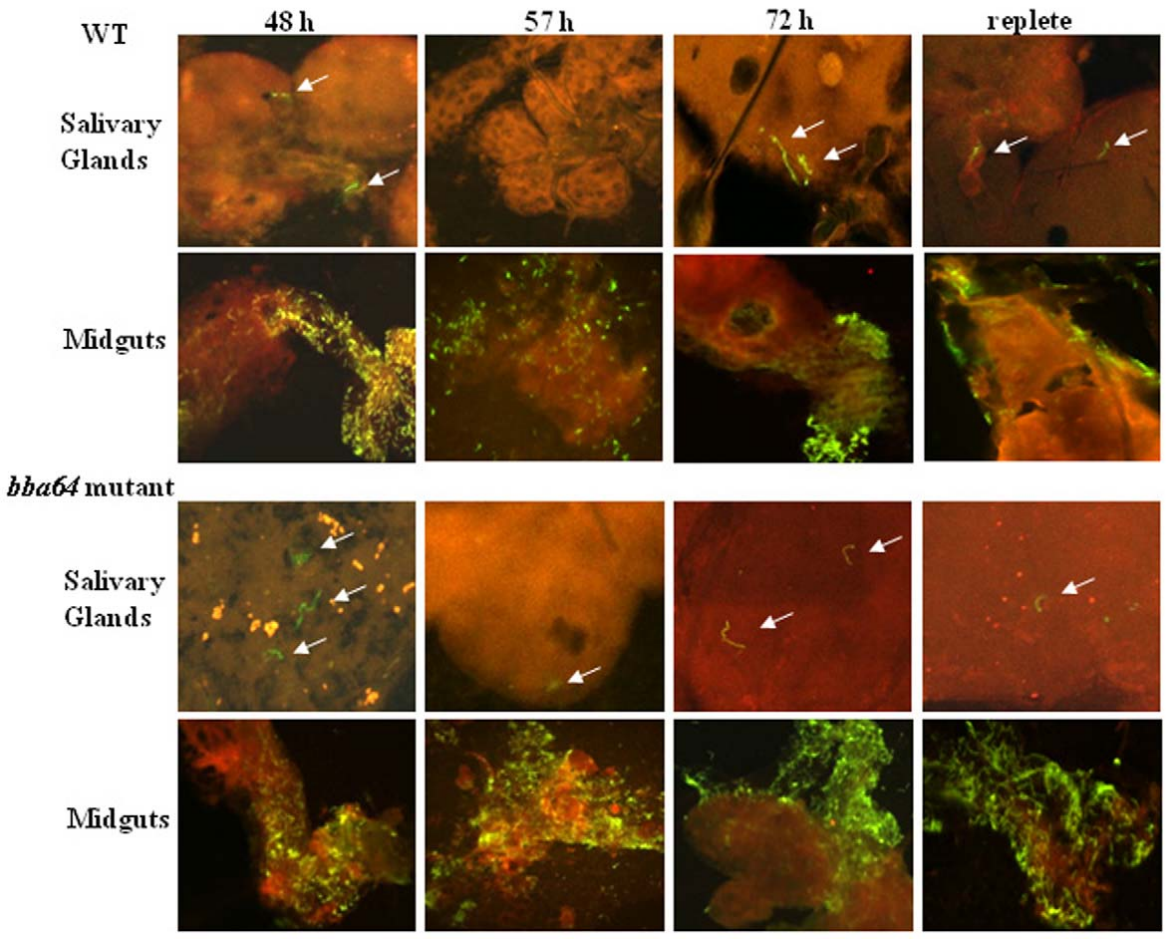
IFA confocal images of nymphal tick salivary glands and midguts. Representative images of salivary glands and midguts removed at 48 h, 57 h, 72 h post and repletion from WT- and *bba64* mutant-infected nymphs. Arrows denote *B. burgdorferi* in salivary glands and the spirochetes in the midguts are represented by the yellow-green color. Magnification of salivary gland fields 400X; magnification of midgut fields 250X. The time point (h) at which the nymph was removed from the mouse is labeled above each composite.

Midguts were excised from actively feeding and replete infected nymphs, and immunofluorescence assays (IFA) were performed to determine the presence of spirochetes. WT and *bba64* mutant spirochetes were visualized in the nymphal midguts at all time points (48, 57, 72 h, and repletion) throughout the feeding (Figure 3). Additionally, the *bba64* mutant was culturable from the nymphal midguts (9/9 midguts culture positive).

### The *bba64* mutant migrates to the salivary glands

IFAs were performed with salivary glands excised from actively feeding and replete infected nymphs to determine the presence of spirochetes at various time points during the nymphal feeding. Both WT and *bba64* mutant spirochetes were observed in the salivary glands, but neither were seen in every salivary gland from individual nymphs at specified times (Figure 3 & Table 1). Although the WT strain was not detected at 57 h in any salivary gland sample, the *bba64* mutant was observed in some excised salivary glands at all time points (Figure 3 & Table 1). Confocal Z-sectioning of the salivary glands provided views suggesting that the organisms were present within the salivary glands and not located externally (Figure 3).

**Table 1:** Immunofluorescent imaging of *B. burgdorferi* in nymphal salivary glands.

Time Point (h)	# salivary glands with WT/total # nymphs	# salivary glands with *bba64* mutant/total # nymphs
48	3/8	2/10
57	0/6	3/7
72	1/7	4/8
replete	2/4	2/4

Salivary glands were cultured from infected nymphs at 57 h, 72 h, and repletion to determine the viability of the *bba64* mutant. The *bba64* mutant was culturable from the salivary glands (8/9 salivary glands culture positive).

### The *bba64* mutant is not culturable from mouse skin

To determine if the bba64 mutant isolate was deposited into the mouse dermis as the infected nymphs feed, we contained infected nymphal ticks within capsules attached to mice, allowed the infected nymphs fed to repletion, and cultured the skin at the bite site. Skin samples from 70% of mice fed upon by WT-infected nymphs were culture positive, while all skin samples from mice fed upon by the *bba64* mutant-colonized nymphs were culture negative (Table 2). Furthermore, 89% of skin samples from mice fed upon by the *bba64* complemented mutant-infected nymphs were culture positive, demonstrating the restoration of the WT phenotype (Table 2). Control skin sites taken for biopsy adjacent and distal to the bite site were all culture negative for each strain.

**Table 2:** *Borrelia burgdorferi* mouse skin biopsy cultures, following feeding of infected nymphs contained within a capsule.

*B. burgdorferi* strain	# culture positive biopsies/# mice challenged
B31-A3 (WT)	7/10
*bba64* mutant	0/10^a^
*bba64* complement	8/9

a statistically significant compared to WT and *bba64* complement, p<0.001.

### The *bba64* mutant does not infect MyD88-deficient mice

To establish if the *bba64* mutant was transmitted from the tick and deposited into the mouse skin but was subsequently eliminated by the innate immune response, we performed nymphal tick feedings on Myeloid Differentiation marker 88 (MyD88)-deficient mice. The MyD88-deficient mice lack a MyD88-dependent innate immune response. The *bba64* mutant was unable to infect the MyD88-deficient or C57BL/6J background control mice when challenged by infected nymphs (Table 3). However, both the WT and *bba64* complemented spirochetes infected 80% of the MyD88-deficient mice and 100% of the control mice when challenged by tick bite (Table 3).

**Table 3:** Feeding of infected nymphs on MyD88 deficient mice.

*B. burgdorferi* strain	# Myd88^−^ mice infected/# challenged^a^	# C57BL/6J mice infected/# challenged^a^
B31-A3 (WT)	4/5	5/5
*bba64* mutant	0/5^b^	0/5^c^
*bba64* complement	4/5	5/5

a Mouse infection status was determined by serology and ear culture.

b statistically significant compared to WT and *bba64* complement, p = 0.018.

c statistically significant compared to WT and *bba64* complement, p≤0.001.

## Discussion

The *bba64* gene was originally recognized to encode a highly immunogenic lipoprotein found amongst a variety of other genes that were differentially expressed in response to environmental parameters that mimic a feeding tick [6], [7], [9], [12], [14], [15], [25]. Our laboratory previously demonstrated that a *bba64* mutant could infect mice by needle inoculation, infect ticks via xenodiagnosis, and persistently colonize larval, nymphal, and adult ticks; however, the *bba64* mutant was unable to infect mice by tick bite transmission [33]. The current study was performed to determine the mechanism by which BBA64 functions to facilitate infection. We analyzed the phenotypic properties of the BBA64-deficient strain within the tick and found that the mutant was not defective in replication or migration into the salivary glands as the nymph fed (Figures 2 & 3). Furthermore, the mutant was not culturable from mouse skin following tick feeding, and was unable to infect MyD88-deficient mice by nymphal infestation. The results from this study bolsters our hypothesis that BBA64 functions to deliver *B. burgdorferi* out of the vector and narrows the location to which this occurs to the salivary gland-host interface.

In this analysis, we measured *bba64* expression temporally during engorgement relative to expression in flat nymphs. qRT-PCR revealed that *bba64* was expressed throughout consumption of the entire bloodmeal and for at least 8 hours after detachment (Figures 1A). The highest level of *bba64* expression was observed after 48 hours of feeding, suggesting that BBA64 is produced in preparation for borrelial transmission into the vertebrate host. Our laboratory reported earlier that *bba64* expression was undetectable in nymphs after 24 hours post detachment, [31]; however, in this study, we observed expression in replete ticks at 8 hours or less post detachment (Figure 1). Collectively, these data indicate that *bba64* expression is upregulated in response to stimuli preparing the *Borrelia* for migration out of the tick, and begins to decrease once the intake of the bloodmeal ceases and the fed nymph detaches from the host.

Sufficient borrelial replication inside the tick's midgut is necessary for transmission. *Borrelia* strains with inactivated *guaAB, bb0690, bb0323, ospA/B, bb0365*, or *bptA* possessed a growth defect inside the vector that resulted in: 1) lower spirochete densities as the tick fed, 2) an inability to persist within ticks, or 3) both [24], [37], [38], [39], [40], [41]. In contrast to the aforementioned *Borrelia* mutant isolates, the *bba64* mutant was able to replicate normally within the feeding nymph (Figure 2). At later time points during the nymphal feeding, the numbers of *bba64* mutant were significantly more abundant inside the engorged tick than of WT (Figure 2). An explanation for this finding is that inactivating *bba64* may result in the retention of more spirochetes in the nymph due to a lack of transmission to the vertebrate host.

A prerequisite for spirochete transmission into the vertebrate host is migration into the tick's salivary glands [42], [43], [44]. In our study, images of salivary glands during consumption of the bloodmeal showed WT and *bba64* mutant spirochetes present, indicating that the mutant was able to migrate to the salivary glands (Figure 3). Although we observed spirochetes in some of the salivary glands collected, and determined that they were viable by culture, we are currently investigating whether the *bba64* mutant exhibits attenuated migration efficiency from midgut to salivary glands. For example, *bba64* inactivation may result in the lack of a critical mass of organisms within the salivary glands required for transmission. We did not attempt qPCR to enumerate *B. burgdorferi* in the extracted salivary glands because when we visualized the organisms in salivary glands by IFA (Fig. 3) the numbers were low and not in all salivary glands, which poses a challenge when determining the number of spirochetes present.

Given that the *bba64* mutant was observed and viable in the salivary glands, we sought to determine if the spirochetes were deposited into the mouse dermis via tick bite. As expected, skin biopsy cultures indicated that the WT and the complemented mutant were readily transmitted to the dermis of naive mice by infected nymphs (Table 2). Also, all skin cultures from mice fed upon by *bba64* mutant-infected nymphs were negative for borrelial growth (Table 2). However, PCR analysis detected DNA in the skin biopsies from all of the borrelial isolates, including the mutant, used in this study (data not shown). Interpretation of the PCR results were inconclusive due to our finding of contaminating DNA in nymphal feces shed on the skin surface as the nymphs feed [45]. Therefore, the skin culture data suggested either 1) the *bba64* mutant was entering the host but was unable to establish an infection, perhaps by a failure to evade the innate immune response or 2) the *bba64* mutant spirochetes were unable to exit the nymph.

To ascertain if the *bba64* mutant could infect mice with impaired innate immunity, we infested MyD88-deficient mice with WT-, complement-, and *bba64* mutant-infected nymphs. MyD88 is a universal adaptor molecule that is utilized by most Toll-like receptors (TLRs) and interleukin-1 (IL-1) in signal transduction pathways to activate the innate immune response against invading microorganisms [46], [47]. Studies have determined that MyD88-deficient mice display a severe defect in host defense against *B. burgdorferi* infection [48], [49]. We found that the *bba64* mutant did not establish an infection in the MyD88-deficient mice via nymphal infestation, unlike the WT and complement isolates (Table 3). These findings indicate that the *bba64* gene product does not assist *B. burgdorferi* in evasion of a MyD88-mediated innate immune response. Notably, a BBA64 innate immune evasion function is inconsistent with our previous finding that intradermal needle-inoculated *bba64* mutants are capable of establishing an infection in mice [33].

Recent studies have identified additional *B. burgdorferi* genes that play a role in facilitating mammalian infection by tick bite. Inactivation of *bba07* resulted in murine infection via needle inoculation but not via tick infestation, whereas knocking out *lp6.6* (*bba62)* and *bba52* implicated a putative role for these genes in impaired pathogen transmission from the vector to the vertebrate host [50], [51], [52]. These genes and *bba64* are localized to lp54, a finding that leads us to infer that several genes on this plasmid encode proteins that may interact and/or be involved in *B. burgdorferi*-tick-host interactions.

In conclusion, we investigated the phenotypic traits of the BBA64-deficient strain within feeding nymphs in an effort to define the function of this *B. burgdorferi* surface protein as it pertains to vector transmission. We demonstrated that BBA64 is not involved in i) borrelial replication during tick engorgement, ii) migration to salivary glands, or iii) evasion of MyD88-dependent innate immunity. Therefore, our results point to the interaction of BBA64 likely occurring in the junction between salivary gland and host deposition. For example, BBA64 may be a receptor for a tick salivary gland protein necessary for transporting *B. burgdorferi* out of the tick into the host. Although the precise mechanism by which the *bba64* gene product causes attenuated infectivity for mice by tick bite remains to be defined, data presented herein significantly advances our understanding for this protein's role in *B. burgdorferi* pathogenesis.

## Materials and Methods

### Ethics statement

This study was carried out in strict accordance with the recommendations in the Guide for the Care and Use of Laboratory Animals of the National Institutes of Health. This protocol was approved by the Institutional Animal Care and Use Committee (IACUC) of the Centers for Disease Control and Prevention, Division of Vector-Borne Diseases, Fort Collins, CO (PHS Assurance #A-4366-01). All procedures were performed as described in the IACUC-approved protocol, and all efforts were made to minimize suffering.

### Bacterial strains, ticks, and mice

*B. burgdorferi* WT clone B31-A3 [53], *bba64* mutant (*bba64*::flgkan, [33]), and *bba64* complemented mutant (*bba64*::flgkan-ciscomp, [33]) strains were grown in Barbour-Stoenner-Kelly II (BSK-II) complete culture medium at 34°C in capped tubes. All *B. burgdorferi* isolates were maintained as low-passage (<2) frozen stocks in 30% glycerol at −80°C and maintained the full complement of plasmids, except for cp9. Infected *I. scapularis* tick colonies were generated via xenodiagnosis by feeding clean *I. scapularis* larva on Swiss-Webster outbred mice previously infected via needle inoculation with 1×10^4^ of WT, *bba64* mutant, or *bba64* mutant complemented isolates [33], [54]. Female Swiss-Webster mice, 6–8 week old, were from a specific pathogen-free colony maintained at the Division of Vector-Borne Diseases, Centers for Disease Control and Prevention (Fort Collins, CO). Female MyD88 deficient (*Myd88^tm1Defr^*/J) and control (C57BL/6J) mice were purchased from Jackson Laboratories (Bar Harbor, MN).

### Nymphal tick feeding time course

Female Swiss-Webster outbred mice were anesthetized with a ketamine (66.667 mg/kg) and xylazine (6.67 mg/kg) mixture and 15 *I. scapularis* nymphs infected with the WT or *bba64* mutant were placed on the mice dorsally between the scapulae and allowed to attach. Nymphs were gently removed with fine-tip forceps at 33, 48, 57, and 72 h post infestation or allowed to feed to repletion. Nymphs from separate feedings were collected and subjected to RNA extraction, salivary gland dissection, or DNA preparation, as described below.

### MyD88 tick feeding

Female MyD88-deficient and C57BL/6J mice were anesthetized as described above, and 10 WT-, 10 *bba64* mutant-, or 15 *bba64* complement-infected nymphs were placed on the mice dorsally between the scapulae and allowed to feed to repletion (approximately 4 d). Mice were assayed for infection 21 d following the nymphal feeding by serology (immunoblotting against whole cell *B. burgdorferi* lysates) and culture of ear biopsies in BSK-II supplemented with antibiotics and fungizone as described previously [55]. Statistical analysis, comparing the number of WT- or *bba64* complement-infected mice to the number of *bba64* mutant-infected mice, was performed using Kruskal-Wallis one-way analysis of variance on ranks and a Tukey test for pairwise comparison.

### Nymphal tick feedings in a capsule

Mice were anesthetized as described above and a small area on the dorsal surface between the scapulae was shaved. A feeding capsule (18 mm in diameter; Nalgene, Rochester, NY) was glued to the skin using a mixture of 3 parts colophony resin (Kramer Pigments Inc., New York, NY) and 1 part beeswax [56]. Ten WT-, *bba64* mutant-, or *bba64* complement-infected nymphal ticks were placed into the capsule, and a small piece of mesh was attached to the top with the colophony-beeswax mixture. The mesh prevented nymphs from escaping the contained area and was removed after 24 h following nymphal placement. After 96 h, the mice were euthanized, the capsules were removed, and any remaining feeding nymphs were detached with forceps. The skin under the capsule was swabbed with 70% ethanol and biopsies were taken at the bite site (within the capsule area), next to the capsule, and at a control site distal from the bite site. Skin biopsies (cut into approx. 1mm^2^ sections) were cultured in BSK-II supplemented with antibiotics and fungizone, incubated for up to 28 d at 34°C in capped tubes, and were analyzed for *B. burgdorferi* growth by dark field microscopy. Statistical analysis was performed comparing the biopsy culture results using Kruskal-Wallis one way analysis of variance on ranks and Dunn's method for pairwise comparison.

### qPCR

Quantification of *B. burgdorferi* in individual nymphs infected with the WT or *bba64* mutant was carried out by using qPCR with TaqMan Universal PCR Master Mix (Applied Biosystems, Austin, TX) and *flaB* TaqMan probe and primers [19]. Actively feeding infected nymphal ticks (5 per time point) were gently removed with forceps at 33, 48, 57, and 72 h and were homogenized individually with glass Tenbroek grinders in a total of 200 µl of phosphate buffered saline (PBS). Replete and flat (0 h) infected nymphs were homogenized in the same manner. Uninfected nymphs were also assayed as a control to detect any non-specific PCR amplification by the *flaB* probe and primers. The homogenized nymphal suspensions were placed in a boiling water bath for 5 min and stored at −20°C. Real-time PCR reactions contained 1 µl homogenized nymphal suspensions, 1 µM 5′ *flaB* primer, 1 µM 3′ *flaB* primer, 0.1 µM *flaB* probe, and 1X TaqMan universal PCR master mixture in a total volume of 25 µl. Amplification conditions included 1 cycle at 95°C for 10 min and 50 cycles of 95°C for 30 sec and 60°C for 1 min, with data collection after each cycle. Amplification of each DNA sample was performed in triplicate. To calculate copies of *flaB*, a standard curve was generated by amplifying *flaB* cloned into pCR4 with the TOPO TA Cloning kit (Invitrogen, Carlsbad, CA). Statistical analysis comparing copies of *flaB* in WT- and *bba64* mutant-infected nymphs was performed using Mann-Whitney rank sum test; n = 5 nymphs.

### RNA isolation and qRT-PCR

RNA was isolated from flat (0 h), replete, and actively feeding (33, 48, 57, and 72 h) WT-infected nymphs. Four WT-infected nymphs were collected and pooled from each time point. Nymphs were homogenized with a glass tenbroek grinder in 500 µl RNAlater (Ambion, Austin, TX) and stored at −80°C. RNAlater was removed from the samples by pelleting the nymphal hemogenates at 16000× g for 5 min and decanting the supernatant. This was followed by total RNA extraction of the pellet using the RNAqueous RNA isolation kit according to the manufacturer's instructions (Ambion). Contaminating DNA was removed using the TURBO DNA-free kit (Ambion) according to the manufacturer's instructions. DNA contamination was tested, in triplicate, using real-time PCR with a *flaB* TaqMan probe prior to cDNA synthesis. Total RNA was quantified using a Nanodrop 2000 spectrophotometer (Thermo Scientific, Rockford, IL).

cDNA was generated using 100 ng total RNA with the RETROscript Kit (Applied Biosystems) and 5 µM random decamers (Ambion) incubated at 44°C for 60 min followed by incubation at 92°C for 10 min to inactivate the reverse transcriptase. Real-time PCR was performed with TaqMan Universal PCR Master Mix (Applied Biosystems) using *flaB* and *bba64* TaqMan probe and primers described previously [19], and *ospC* probe (5′-TGTGAAAGAGGTTGAAGCGTTGCTGTC-3′) and primers (forward 5′-CGGATTCTAATGCGGTTTTACTTG-3′ and reverse 5′-CAATAGCTTTAGCAGCAATTTCATCT-3′) using a Bio-Rad iCycler (Bio-Rad, Hercules, CA). Real-time PCR reactions, performed in triplicate, contained 1 µl cDNA, 1 µM 5′ primer, 1 µM 3′ primer, 0.1 µM probe, and 1X TaqMan universal PCR master mixture in a total volume of 12.5 µl. Amplification conditions included 1 cycle at 95°C for 10 min and 50 cycles of 95°C for 30 sec and 60°C for 1 min, with data collection after each cycle. *bba64* and *ospC* expression were determined relative to the levels in WT-infected flat nymphs using the 2^-ΔΔ CT^ method [57], and were normalized to the constitutively expressed *flaB*. qRT-PCR was unable to detect *bba64* and *ospC* in flat nymphs (time 0); therefore, flat nymphs were assigned a crossing threshold (C_T_) value of 50 for analysis. The iCycler software determined crossing threshold (C_T_) values for all other timepoints. Data was calculated from average of 4 independent nymphal feedings for each time point (each technically replicated in triplicate). Statistically analysis comparing *bba64* expression to baseline expression (0) was performed using Mann-Whitney rank sum test, n = 4.

### Immunofluorescence labeling

Salivary glands and midguts were removed at different time points throughout the feeding from the WT- and *bba64* mutant-infected nymphs. Salivary glands were washed several times in fresh drops of PBS on a microscope slide. Midguts and washed salivary glands were placed in separate drops of PBS on silane-treated glass slides (BioWorld, Dublin, OH), dried, and fixed with acetone for 10 min. To block nonspecific binding the slides were incubated with 10% bovine serum albumin (BSA) in PBS at room temperature for 30 min, and then stained with fluorescein isothiocyanate (FITC)-conjugated rabbit anti-*B. burgdorferi* (GenWay Biotech Inc., San Diego, CA) for 1 hr at 37°C in a humidified chamber. After incubation the slides were washed (3 X PBS) and cover slips were mounted with ProLong Gold antifade reagent (Invitrogen, Eugene, OR). The spirochetes inside the midguts and salivary glands were viewed with a Zeiss LSM 5 Pascal confocal laser scanning microscope with 250X and 400X magnification, respectively. Confocal images were analyzed using the LSM 5 image browser (Carl Zeiss Inc., New York, NY).
